# Inter-nesting movements and habitat-use of adult female Kemp’s ridley turtles in the Gulf of Mexico

**DOI:** 10.1371/journal.pone.0174248

**Published:** 2017-03-20

**Authors:** Donna J. Shaver, Kristen M. Hart, Ikuko Fujisaki, David Bucklin, Autumn R. Iverson, Cynthia Rubio, Thomas F. Backof, Patrick M. Burchfield, Raul de Jesus Gonzales Diaz Miron, Peter H. Dutton, Amy Frey, Jaime Peña, Daniel Gomez Gamez, Hector J. Martinez, Jaime Ortiz

**Affiliations:** 1 National Park Service, Padre Island National Seashore, Corpus Christi, Texas, United States of America; 2 U.S. Geological Survey, Wetland and Aquatic Research Center, Davie, Florida, United States of America; 3 University of Florida, Ft. Lauderdale Research and Education Center, Davie, Florida, United States of America; 4 Cherokee Nation Technologies, contracted to U.S. Geological Survey, Wetland and Aquatic Research Center, Davie, Florida, United States of America; 5 Gladys Porter Zoo, Brownsville, Texas, United States of America; 6 Acuario de Veracruz A.C., Veracruz, Veracruz, Mexico; 7 National Oceanographic and Atmospheric Administration, National Marine Fisheries Service, Southwest Fisheries Science Center, La Jolla, California, United States of America; Auburn University, UNITED STATES

## Abstract

Species vulnerability is increased when individuals congregate in restricted areas for breeding; yet, breeding habitats are not well defined for many marine species. Identification and quantification of these breeding habitats are essential to effective conservation. Satellite telemetry and switching state-space modeling (SSM) were used to define inter-nesting habitat of endangered Kemp’s ridley turtles (*Lepidochelys kempii*) in the Gulf of Mexico. Turtles were outfitted with satellite transmitters after nesting at Padre Island National Seashore, Texas, USA, from 1998 through 2013 (n = 60); Rancho Nuevo, Tamaulipas, Mexico, during 2010 and 2011 (n = 11); and Tecolutla, Veracruz, Mexico, during 2012 and 2013 (n = 11). These sites span the range of nearly all nesting by this species. Inter-nesting habitat lies in a narrow band of nearshore western Gulf of Mexico waters in the USA and Mexico, with mean water depth of 14 to 19 m within a mean distance to shore of 6 to 11 km as estimated by 50% kernel density estimate, α-Hull, and minimum convex polygon methodologies. Turtles tracked during the inter-nesting period moved, on average, 17.5 km/day and a mean total distance of 398 km. Mean home ranges occupied were 725 to 2948 km^2^. Our results indicate that these nearshore western Gulf waters represent critical inter-nesting habitat for this species, where threats such as shrimp trawling and oil and gas platforms also occur. Up to half of all adult female Kemp’s ridleys occupy this habitat for weeks to months during each nesting season. Because inter-nesting habitat for this species is concentrated in nearshore waters of the western Gulf of Mexico in both Mexico and the USA, international collaboration is needed to protect this essential habitat and the turtles occurring within it.

## Introduction

The reproductive period is a time of increased vulnerability for many marine and terrestrial animal species. Animals that congregate in specific breeding areas are at particular risk, especially when threats to them are also concentrated in those areas. Sea turtles, salmonids, and many marine mammal species migrate to specific breeding areas through natal homing, imprinting, social facilitation, or other navigational mechanisms [1–3]. Their return to these areas through successive generations maintains these breeding areas through time and ties their reproductive success and population status to these areas. One example is the Kemp’s ridley sea turtle *Lepidochelys kempii*, the most endangered sea turtle species in the world. There is only one genetic stock [4] and one regional management unit (RMU; [5]) for this species. Virtually all Kemp’s ridley nesting occurs along the shores of the western Gulf of Mexico (GOM), with about 97% in Tamaulipas, Mexico, 2% in Veracruz, Mexico (VC), and 1% in Texas, USA. Only scattered nesting occurs elsewhere in the USA [6]. The primary nesting beach for the species is Rancho Nuevo, Tamaulipas (RN 23.180^o^ N, 97.797^o^ W) [7]. The epicenter of nesting in the USA is Padre Island National Seashore (PAIS), Texas, where a bi-national effort has been undertaken to form a secondary nesting colony as a safeguard for this species in the advent of a political or environmental disaster at the primary nesting beach [8].

Conservation efforts, on-going to help recover this species since the mid-1960s, were showing promising signs of success until recently [9]. After reaching a low of only 702 nests worldwide in 1985, nesting increased exponentially through 2009, but declined during 2010 (the year of the Deepwater Horizon Oil Spill; [10,11]), and although it rebounded to near-2009 levels during 2011 and 2012, nesting was below what would have been predicted if the recovery rate had remained exponential. Further, nesting was comparatively reduced during 2013 through 2015. These unexpected changes in the exponential trajectory of nesting have rekindled concern about species recovery [8,12,13].

Kemp’s ridley turtles mature when 10–15 years old, remigrate at approximately two year intervals, and nest an estimated 2–3 times during a nesting season, with consecutive nestings occurring about 21 to 25 days apart [9,14,15]. Since nearly all Kemp’s ridley nesting is restricted to the western GOM [9], a substantial portion of all adults of this species reside there during the breeding season. Reproductively active sea turtles are of the highest value to the population, and their survival is extremely important to population growth and recovery [16]. Because they are concentrated in breeding habitats for months at a time, breeding adults are particularly vulnerable to human related and natural threats that occur there. While incidental capture by shrimp trawling and other fisheries are considered the most significant threat to the species, oil and gas exploration, oil spills, shipping, boat strikes, dredging, hypoxia, and other factors also pose potential harm [9,17].

Although important foraging areas and migratory corridors have recently been identified for adult Kemp’s ridley turtles in nearshore waters of the GOM [18–23], limited information is available on inter-nesting movements and habitats [22–24]. To develop effective management strategies to protect adult females in the marine environment, it is vital to identify their inter-nesting areas in the USA and Mexico [9,25] so that overlap with threats can be quantified. This information can then aid in the delineation of protected areas and development of conservation strategies. For other sea turtle species, satellite tracking data have been used to identify inter-nesting movements and habitat, assess threats within those habitats, and assist with marine spatial planning for conservation in the USA and other countries [26–34]. Here we characterize inter-nesting habitat in the GOM for Kemp’s ridleys tracked by satellite telemetry after nesting at or near PAIS, RN, and VC. Objectives of our study were to: (1) spatially define the inter-nesting habitat; (2) define bathymetry and distance from shore of the inter-nesting habitat; and (3) examine the spatial overlap of threats within that inter-nesting habitat.

## Materials and methods

### Study sites

**Fig 1:**
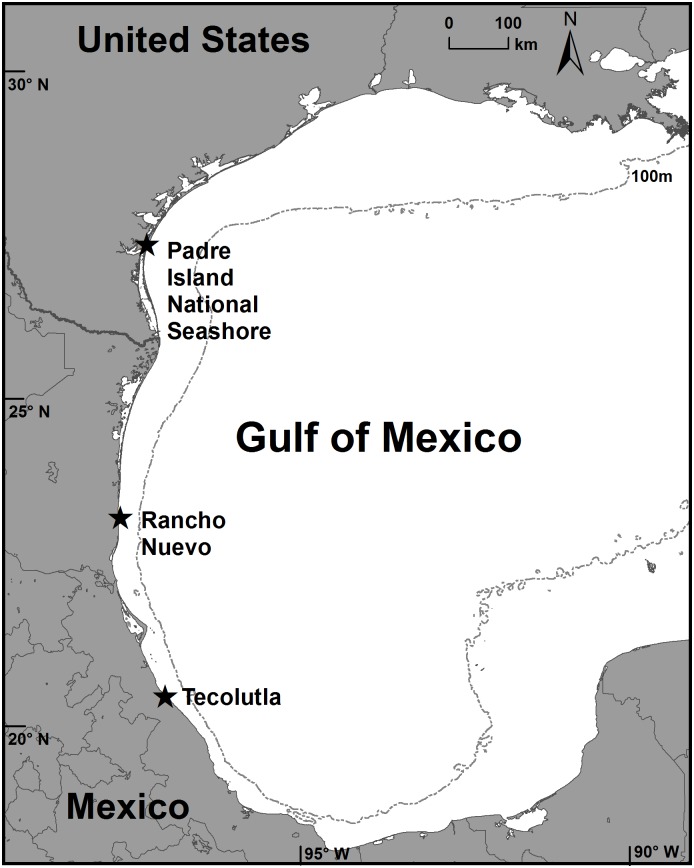
Tagging locations for n = 82 Kemp’s ridley turtles tracked by satellite telemetry in the Gulf of Mexico. Includes n = 60 tracked after nesting at Padre Island National Seashore, Texas, USA during 1998 through 2013, n = 11 at Rancho Nuevo, Tamaulipas, Mexico during 2010 and 2011, and n = 11 at Tecolutla, Veracruz, Mexico during 2012 and 2013.

We tracked turtles from three study sites in the western GOM (Fig 1). The Texas, USA site, the northern extent of the documented historic nesting range for Kemp’s ridley turtles, includes the northernmost 50 km of PAIS, managed by the USA National Park Service. The Tamaulipas, Mexico site, the epicenter of nesting for the species, includes 30 km of beachfront at RN, of which 25 km is managed as a natural reserve. The VC site, the southern extent of the nesting range, includes 50 km of beachfront at and near Tecolutla, which is managed by the national park system in Mexico and a non-governmental organization. These locations are separated by approximately 757 km (straight line distance).

### Turtle capture and transmitter deployment

Kemp’s ridley turtles nest from approximately April through mid-July in Texas and mid-March through August in Tamaulipas and Veracruz. Daily daytime surveys were conducted through the nesting seasons in these areas. Females were intercepted and outfitted with platform transmitter terminals (PTTs) after they nested. Eighty-two PTTs were deployed on Kemp’s ridleys that nested at PAIS from 1998 through 2013 (n = 60); Rancho Nuevo during 2010 and 2011 (n = 11); and Tecolutla during 2012 and 2013 (n = 11) (see S1 Table for PTT models and duty cycles used). These PTTs were deployed on 70 individuals, including 60 tracked one time, eight tracked two times, and two tracked three times; those tracked multiple times were all from the PAIS study site. Each turtle was documented, measured for straight carapace length (SCL), tagged with one passive integrated transponder (PIT) tag and two Inconel flipper tags, and outfitted with a PTT using established protocols [21,35].

### Permitting and animal welfare

This study was carried out in strict accordance with the recommendations in the Guide for the Care and Use of Laboratory Animals of the National Institutes of Health. Work at PAIS was authorized under U.S. Fish and Wildlife Service Permit TE840727-3 and Texas Parks and Wildlife Department Scientific Permit SPR-0190-122, and performed in accordance with Institutional Animal Care Protocol NPS IACUC 2011–15. Work at RN was authorized under USFWS Agreement No. 20181-A-J819, TPWD Permit SPR-0790-004, and Secretaria de Medio Ambiente y Recursos Naturales, Subsecretaria de Gestion para la Proteccion Ambiental, Direccion General de Vida Silvestre Permiso No. SGPA/DGVS/03990/11. SEMARNAT-Direccion General de Vida Silvestre Permiso No. SGPA/DGVS/05559/14 authorized work by personnel in VC.

### Sea turtle tracking

Satellite Tracking and Analysis Tool (STAT; [36]), available at www.seaturtle.org, was used to compile PTT location data. Argos performed traditional least-squares location processing (during 1998 through 2010; [37]) and Kalman-filtering (starting in 2011; [38]) of location data. The Kalman-filtering algorithm provides more estimated positions and significantly improves position accuracy [39], particularly for LCs A and B [40]. We used Location Classes (LC) 3, 2, 1, 0, A, and B, and rejected LCZ locations (see S1 Table for Argos assigned accuracy estimates for each LC).

### Switching SSM and inter-nesting locations

We attempted to use switching state-space modeling (SSM) to characterize the inter-nesting movements of the females that were outfitted with PTTs. SSM has been used to identify modes of movement and quantify habitat usage for a variety of marine animals tracked using satellite telemetry [41–50], including inter-nesting movements and habitats for sea turtles [29,30,51]. SSM has two components that account for location errors (observation error based on LC from Argos data) and animal behavior [46,52–55]. The two-state switching correlated random walk models the change between two behavioral states [55–57]. Because we deployed the PTTs during the nesting season, we defined the SSM behavioral modes as either ‘inter-nesting/foraging’ (signified by restricted movement) and ‘migration’ (signified by more directed movement) [29,44].

We used SSM to identify inter-nesting locations for 43 of the PTTs, with data collected until transmitters stopped or through the end of the nesting season (designated as 31 August). The other n = 39 data sets were not robust enough for the model because they contained gaps in tracking; most of these 39 were from the earlier PTTs deployed at PAIS. We estimated SSM model parameters with Markov Chain Monte Carlo (MCMC), following Breed et al. [46], using WinBUGS via the software program R v2.15.2 [58]. Using two independent and parallel chains of MCMC, we estimated the location and behavioral mode every eight hours for each track. We based posterior distribution samples on 10,000 iterations after a burn-in of 7000 and thinned the samples by five. As suggested by Breed et al. [46], we monitored the convergence by observing model parameters for two independent chains mixed in the trace plots.

We used SSM output to assign behavioral modes to the Argos data points for each of the 43 turtle tracks with successful SSM runs. Dates for behavioral modes were determined by the SSM, but we filtered Argos locations within inter-nesting periods corresponding to those dates for analyses. We define an inter-nesting period as a set of consecutive inter-nesting locations for an individual not interrupted by another mode. If a turtle had multiple inter-nesting periods in a season, they were considered as separate tracks. These periods were discriminated from foraging periods as being prior to migration away from nesting beaches. For the 39 non-SSM tracks, we visually examined each track, and defined the inter-nesting period as beginning on the date when the first valid Argos data point was recorded, and ending on the date of the last valid Argos data point prior to a period of directional movement (i.e., an interpreted migration period) away from nesting beaches and towards foraging areas or other nesting areas. For tags for which all valid Argos data points were within the original nesting area (n = 5, all from PAIS), an inter-nesting end date of 29 June (of the nesting year) was applied, which was the latest date identified through the standard non-SSM method for the PAIS turtles.

As the majority of inter-nesting points were close to the shoreline and nearby islands, and considering the spatial error associated with Argos points [59], we “land filtered” the points by excluding only those land points further than one km from the ocean using ArcGIS 10 [60]. Retaining points falling within the one km ocean buffer allowed us to preserve a significant amount (15.5%) of total inter-nesting points, maintaining more of the spatial structure of inter-nesting tracks. We also filtered locations by excluding those at depths greater than 100 m (considered to be biologically implausible) or those requiring swim speeds > 5 km h^-1^ (considered to be unrealistically high) [21,23]. Filtered tracks were used to reconstruct routes.

### Inter-nesting area fidelity

We quantified inter-nesting area fidelity by comparing each turtle’s filtered track during the inter-nesting period to 100 correlated random walks, which were parameterized to use the distances and angles of the true track in a randomized order. We calculated the mean squared distance (r^2^) of actual track relocations and compared it to the distribution of r^2^ values for the 100 replicate track relocations using a Monte Carlo test; turtles were judged to exhibit site fidelity when p > 0.95. We constrained random walks to be within the 100 m bathymetry contour along the GOM (plus a one km buffer) which follows the same rules used for filtering locations. Random walks were generated using the R package *adehabitatLT* [61], and tests completed using *ade4* [62].

### Inter-nesting home ranges

For inter-nesting tracks with five or more locations, we used two methods to estimate inter-nesting home ranges–minimum convex polygon (MCP) and α-Hull. The MCP is a simple home range estimation which encompasses all input points with the smallest possible convex polygon. The α-Hull method for home-range estimation, as described by Burgman and Fox [63], is a refinement of the MCP home range which objectively removes distant areas with fewer observations and has been used previously for home range estimation in sea turtles [64–66]. The α-Hull method creates a Delaunay triangulation network between input points as a starting point (the outer edge of which is identical to the MCP), and removes triangles from the network if their mean edge length exceeds the mean of all edge lengths in the Delaunay triangulation multiplied by a user-defined α. After a preliminary visual analysis to compare different α values at every 0.25 interval from 1 to 5, we chose a value of 1.5, which resulted in α-Hull refinement of the MCP for all but five inter-nesting tracks (all with low sample sizes), and on average removed 14.3% of Delaunay triangles (i.e., those with the largest perimeters). Following removal of triangles, all remaining triangles are combined to represent the home range. For turtles with longer inter-nesting periods (where mean daily locations could be calculated for ≥ 20 days) we also calculated home ranges using a kernel density estimation (KDE; 95% KDE = home range and 50% KDE = core use area) [67–71], with the mean daily locations as input and a smoothing parameter (h) calculated with Least Square Cross Validation (LSCV). We also calculated the ratio of the standard deviation (SD) of lengths in the X and Y dimensions for each set of input points to determine if tracks were skewed in either dimension, and used this ratio to ‘rescale’ the outputting KDE when the ratio was < 0.5 or > 2. MCP and KDE analyses were implemented using the R package *adehabitatHR* [61]; α-Hulls were created using the R packages tripack [72] and spatstat [73] to create the Delaunay triangulations. We created both 100% MCP and α-Hull estimates for all tracks and KDEs in addition to these when possible. All three methods have been used previously in sea turtle studies, though rarely all at once; we used all three methods to enable comparison of our findings across different studies.

### Inter-nesting area characteristics

To characterize the at-sea inter-nesting areas used by individual turtles, we calculated the centroid of each turtle’s 50% KDE/MCP/α-Hull. We summarized centroid distance to the nearest land as well as centroid depth, using bathymetry data from the NOAA National Geophysical Data Center (GEODAS) ETOPO1, 1-arc-minute global relief model of Earth’s surface (http://www.ngdc.noaa.gov/mgg/global/global.html; accessed 26 January 2012). We compared median MCP area for SSM and non-SSM turtles using Mann-Whitney U Statistic. We also compared median MCP area for SSM tracks from the three nesting beaches using Dunn’s Method All Pairwise Multiple Comparison Procedure.

We further quantified and depicted usage of the inter-nesting area by calculating the number of turtle-tracking days and the number of turtles in grid cells (10 x 10 km) for the 43 turtles with SSM tracks and the 39 non-SSM tracks over all years. The grid extended across the inter-nesting habitat in the western GOM within the 100 m isobaths. We counted the number of days each turtle was observed (turtle-days) in each grid cell using all filtered locations except LCZ during inter-nesting periods. For the number of turtles in grid cells, we calculated the number with MCPs, α-Hull, and 95% KDEs for home range in each cell. If a turtle had more than one MCP, α-Hull, or KDE, these were merged so that every turtle was only counted once throughout all grid cells in depictions for MCP, α-Hull, or KDE.

To explore the relationship of distance to shore to inter-nesting habitat selection, we compared the number of turtle-days per distance category (< 5.0 km, 5.0–9.9 km, 10.0–14.9 km, 15.0–19.9 km, 20.0–24.9 km, 25.0–29.9 km, ≥ 30 km) to a hypothetical even distribution of turtle-days within these categories using Chi square analysis. We compared the proportion of MCP distance to shore centroid locations within these categories for SSM and non-SSM turtles using Chi-square analysis. We compared the median MCP centroid distance to shore for SSM turtles versus non-SSM turtles using a Mann-Whitney U Statistic. Pearson Product Moment Correlation was used to test if size of the turtles was related to mean distance from shore values per MCP centroid. Kruskal-Wallis One Way ANOVA on ranks was performed to examine if median MCP centroid distance to land differed between SSM turtles from the three nesting beaches.

To explore the relationship of bathymetry to inter-nesting habitat selection, we compared the number of turtle-days per depth category (≤ 15 m, 15–24 m, 25–34 m, 35–49 m, ≥ 50 m) to a hypothetical even distribution of turtle-days within these categories using Chi square analysis. We compared the proportion of MCP depth centroid locations within these categories (except the ≥ 50 m depth category, which contained no centroids) for SSM and non-SSM turtles using Chi-square analysis. We compared the median MCP centroid depth for SSM versus non-SSM turtles using Mann Whitney U Statistic. Pearson Product Moment Correlation was used to test if size of the turtles was related to mean bathymetry values per MCP centroid. Kruskal-Wallis One Way ANOVA on ranks was performed to examine if median MCP centroid depths differed between SSM turtles from the three nesting beaches. For all statistical comparisons, we used alpha level of 0.05.

Using filtered satellite location data, we calculated the total distance moved (TDM) by each turtle in the inter-nesting period (i.e., from capture date to the last inter-nesting location as defined by SSM for SSM turtles, or from capture date to the date at which directional movement towards foraging areas was observed for non-SSM turtles) by adding up the straight-line distances between successive filtered points. We also calculated an average TDM per day by dividing the TDM per turtle by the number of days in the inter-nesting period for that turtle.

### Identification of nests and nest-site fidelity

We documented successive nests for several of the tracked turtles within and across inter-nesting seasons. Nesting was recorded when observed in Texas [8] or Mexico [9], but nesting turtles were only observed at a portion of nest sites documented during the study years. For years 2002 through 2013, we also used genetic kinship analysis to match nests of unknown maternity found in Texas to nesters documented there (see [74]). We pooled nest records for tracked turtles by including those genetically matched to them and those directly observed. Subsequent nest records were more limited for Mexico nesters due to a lower direct observation rate, and lack of genetic matching for nests of unknown maternity there. For these recaptured tracked turtles, we calculated the distances between successive nests and the mean length of inter-nesting intervals. We classified whether a turtle displayed nest-site fidelity based on the mean distance between successive nests that were recorded (≤ 13.5 km from previous nest = nest-site fidelity; > 13.5 km from previous nest = no nest-site fidelity); nest site fidelity had not been previously defined for Kemp’s ridley turtles, so we based our definition on the median distance between nests (13.5 km, n = 28). We used a Mann-Whitney U Statistic to test if there was a difference between TDM by turtles with nests less than and greater than a mean of 13.5 km apart. We used Pearson Product Moment Correlation to determine if distance between successive nest sites was correlated with turtle size and TDM. For all statistical comparisons, we used alpha level of 0.05.

To display examples of three different types of behavior during the inter-nesting period, in terms of nest site and in-water habitat fidelity, we also filtered data for example tracks to exclude locations that required turning angles < 25° (often indicative of erroneous locations: [59,65], and plotted those tracks and corresponding nests. This additional turning angle filtering helped simplify tracks in order to better display behavioral differences.

### Potential overlap with anthropogenic activities

To examine threats within inter-nesting habitat, we also mapped the overlap of commercial shrimp trawling in the USA during May through August of 2010–2013 and the locations of active oil and gas platforms. We created a layer in ArcGIS 9.3 [75] using shrimp trawling data provided by NOAA (Jim Nance, pers. comm.). The layer for oil and gas platforms in the USA (through 2014) was obtained from http://www.data.boem.gov/ and in Mexico (through 2012) was obtained from https://www.fas.org/sgp/crs/row/R43313.pdf; platforms with a past removal date were removed from the layer before mapping. We described these potential threats for each centroid by providing the number of shrimp trawling effort days associated with the area containing the centroid, and totaling the number of active oil and gas platforms within a 15 km buffer of each centroid. For all statistical analyses, we used SigmaPlot 13.0 [76].

## Results

### Turtles

Mean straight carapace length of the turtles (n = 82) at the time of PTT deployment was 63.1 cm (SCL, ± 2.4 SD; S2 Table and S1 Fig). The 82 PTTs yielded a total of 8937 turtle-tracking days and a mean individual tracking duration of 109.0 d (± 29.3 SD, range 8 to 146 d). Of these 82, 10 individuals were tracked after two (n = 8) and three (n = 2) successive nesting years.

### Nests and nest-site fidelity

For the 60 PTTs deployed on turtles at PAIS, 58 were after their first, one after their second, and one after their third recorded nests of the season; comparable information is not available for PTTs deployed in Mexico due to more limited mark recapture tag monitoring there. Only nests recorded through mark recapture and kinship analysis, from PTT deployment through the end of that nesting season, were included in this analysis. Nests prior to the nesting event associated with tagging were excluded as tracking data were not available for pre-PTT deployment times. Kemp’s ridleys were documented nesting from 1 to 3 times (mean ± SD = 1.4 ± 0.6 nests) from the date of PTT deployment through the end of nesting season. Thirty-one turtles were encountered nesting more than once during the nesting season when tracking was underway in Texas or Mexico, including 26 recorded twice, and five recorded three times. Of the 36 subsequent nests recorded, 30 were from mark recapture tagging observations (n = 27 at PAIS, n = 2 at RN, n = 1 at VC) and six from kinship analyses (all at PAIS). For the 31 turtles, the mean interval between recorded nests was 25.9 ± 11.7 d (range 15 to 78 d). Distances between nests were only available for the PAIS re-nesters; for these 28 turtles, mean straight-line distance between nests ranged from 0.3 to 77.3 km (mean ± SD = 18.7 ± 18.7 km) (S2 Fig). Distance between successive nest sites was not correlated with turtle size (Pearson Product Moment Correlation r = -0.344, p = 0.073, n = 28) or with TDM during the inter-nesting period (Pearson Product Moment Correlation r = -0.231, p = 0.236, n = 28).

There was no significant difference between TDM by turtles with nests less than and greater than 13.5 km apart (Mann-Whitney U Statistic = 79.000, T = 200.000, n(≤ 13.5 km) = 14, n(> 13.5 km) = 14, p = 0.395). Turtles with nests less than 13.5 km apart moved on average 597.1 km (SD = 405.6 km, range 91–1408 km, n = 14). Turtles with nests more than 13.5 km apart moved on average 491.2 km (SD = 383.1 km, range 55.5–1161.3 km, n = 14).

### In-water, inter-nesting areas and periods

Of the 43 turtles with SSM, 32 displayed only one inter-nesting period, whereas 11 showed multiple inter-nesting periods (range = 2 to 4 periods) (Tables 1 and 2, S2 and S3 Tables). For these 11 turtles with multiple centroids during a nesting season, distance between centroids decreased with each successive centroid, with centroids 1–2 from 23.2–369.3 km apart (mean ± SD = 102.7 ± 98.1 km, n = 11), centroids 2–3 from 14.3–14.5 km apart (mean ± SD = 14.4 ± 0.1 km, n = 2), and centroids 3–4 8.0 km apart (n = 1) (Table 2).

**Table 1:** Mean and standard deviation of Kernel Density Estimation (KDE), Minimum Convex Polygon (MCP), and α-Hull results for nesting Kemp’s ridley turtles (*Lepidochelys kempii*) with successful and unsuccessful State-Space Model (SSM) runs.

	KDE				MCP			α-Hull		
	50% area (km^2^)	50% centroid depth (m)	50% centroid to land (km)	95% area (km^2^)	Area (km^2^)	Centroid depth (m)	Centroid to land (km)	Area (km^2^)	Centroid depth (m)	Centroid to land (km)
**Successful SSM runs**										
***Padre Island National Seashore*, *Texas*, *USA***										
Mean	344.7	14.0	5.0	1272.7	903.3	16.7	8.6	358.0	13.8	5.6
SD	113.6	2.6	1.9	323.6	1323.6	6.4	5.8	603.8	5.3	4.4
N = 35 centroids, 25 turtles										
***Rancho Nuevo*, *Tamaulipas*, *Mexico***										
Mean					183.6	12.4	4.7	48.6	7.6	2.4
SD					103.6	9.5	3.2	36.1	4.9	1.4
N = 10 centroids, 6 turtles										
***Tecolutla*, *Veracruz*, *Mexico***										
Mean	46.1	2.7	2.3	236.3	1035.1	17.0	7.2	192.9	5.5	3.6
SD	2.6	1.2	0.6	54.0	940.6	14.0	3.6	164.5	5.0	2.4
N = 11 centroids, 11 turtles										
***Overall SSM***										
Mean	195.4	8.3	3.7	754.5	800.7	16.0	7.6	270.3	11.0	4.6
SD	178.7	6.5	1.9	604.4	1154.3	8.9	5.2	495.5	6.2	3.9
**Unsuccessful SSM runs**										
***Padre Island National Seashore*, *Texas*, *USA***										
Mean	1175.2	16.7	6.9	3825.9	3841.1	25.3	18.0	1536.6	20.4	11.8
SD	1009.2	4.5	4.2	2706.2	2503.2	6.5	8.1	1246.4	5.8	7.0
N = 33 centroids, 33 turtles										
***Rancho Nuevo*, *Tamaulipas*, *Mexico***										
Mean					400.7	17.7	6.9	284.0	22.3	7.9
SD					421.5	7.8	3.2	379.2	14.6	4.6
N = 3 centroids, 3 turtles										
***Overall Non-SSM***										
Mean	1175.2	16.7	6.9	3825.9	3554.4	24.7	17.1	1432.2	20.6	11.5
SD	1009.2	4.5	4.2	2706.2	2582.4	6.9	8.4	1245.7	6.6	6.9
**Successful and Unsuccessful runs**										
***Overall***										
Mean	895.3	14.3	6.0	2948.4	1878.2	19.4	11.4	725.0	14.8	7.3
SD	962.6	6.3	3.9	2690.6	2279.5	9.2	8.1	1034.6	7.9	6.2
N = 92 centroids, 78 turtles										

**Table 2:** Distances between successive Minimum Convex Polygon (MCP) centroids for 11 nesting Kemp’s ridley turtles (*Lepidochelys kempii*) that had multiple centroids during the same nesting season.

Tag no.	Tagging location^a^	Inter-nesting period for MCP (days)	No. of tracking days	No. of centroids	Distance centroid 1–2 (km)	Distance centroid 2–3 (km)	Distance centroid 3–4 (km)	Total distance (km)
7689	PAIS	4/30/1998–5/22/1998	23	2	156.4			156.4
70700	PAIS	5/1/2007–5/19/2007	19	2	71.9			71.9
82215	PAIS	5/5/2008–6/4/2008	31	2	54.5			54.5
101136	PAIS	4/7/2011–4/21/2011	15	2	131.8			131.8
101137	PAIS	4/22/2011–5/7/2011*	16	3	99.3	14.3		113.6
101139	PAIS	4/23/2011–6/9/2011	48	4	27.1	14.5	8.0	49.5
106341	PAIS	4/28/2011–5/24/2011	26	2	23.2			23.2
100391	RNMX	5/18/2011–6/8/2011	22	2	29.4			29.4
100394	RNMX	5/7/2011–6/6/2011	31	2	93.1			93.1
100395	RNMX	5/2/2011–6/7/2011	37	2	73.9			73.9
100403	RNMX	6/11/2011–6/29/2011	19	2	369.3			369.3

^**a**^Tagging location = PAIS = Padre Island National Seashore, Texas, USA; RNMX = Rancho Nuevo, Tamaulipas, Mexico.

*Did have inter-nesting start on 4/18/2011, but this first inter-nesting period did not have enough data for a home range.

Overall inter-nesting seasons for individuals (from capture date to last inter-nesting point regardless of migration periods) totaled 1838 days and ranged from 2 to 106 days (mean ± SD = 22.4 ± 17.9 d, n = 82). For some, the inter-nesting period was only a few days because the turtles soon migrated from the area after being outfitted with PTTs. In contrast, the inter-nesting period was longer for others because they remained off the nesting beach before migrating away from the area, typically because they nested again that season. Overall inter-nesting seasons for individuals defined by SSM for turtles totaled 863 days and ranged from 2 to 106 d (mean ± SD = 20.1 ± 17.0 d, n = 43), and for non-SSM turtles totaled 975 days and ranged from 2 to 78 d (mean ± SD = 25.0 ±18.8 d; n = 39). The number of days during the inter-nesting period were not significantly different for SSM versus non-SSM tracks (Mann-Whitney U Statistic = 716.000, T = 1741.0000, n(non-SSM) = 39, n(SSM) = 43, p = 0.257).

### Home range and core use area estimates

**Fig 2:**
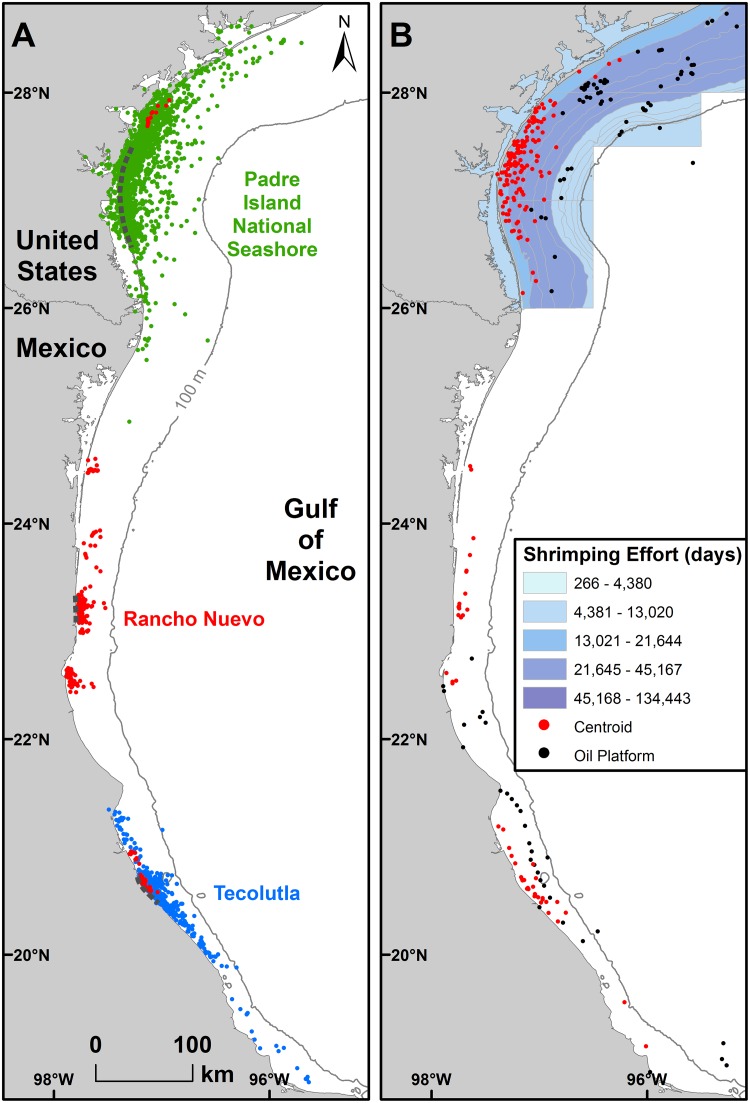
Inter-nesting habitat for Kemp’s ridley turtles tracked after nesting. Includes n = 60 tracked after nesting at Padre Island National Seashore, Texas, USA during 1998 through 2013, n = 11 at Rancho Nuevo, Tamaulipas, Mexico during 2010 and 2011, and n = 11 at Tecolutla, Veracruz, Mexico during 2012 and 2013. (A) Mean daily locations for turtles tracked during inter-nesting periods (n = 82) (B) Kernel density estimates (KDE) centroids and minimum convex polygon (MCP) centroids (red dots) (n = 92) in relation to active oil and natural gas platforms (black dots) in the USA (through 2014; data from http://www.data.boem.gov) and Mexico (through 2012; data from https://www.fas.org/sgp/crs/row/R43313.pdf) and shrimp trawling effort in the USA (days; data provided by NOAA) from May through August of 2010–2013; data were not available for Mexico.

**Fig 3:**
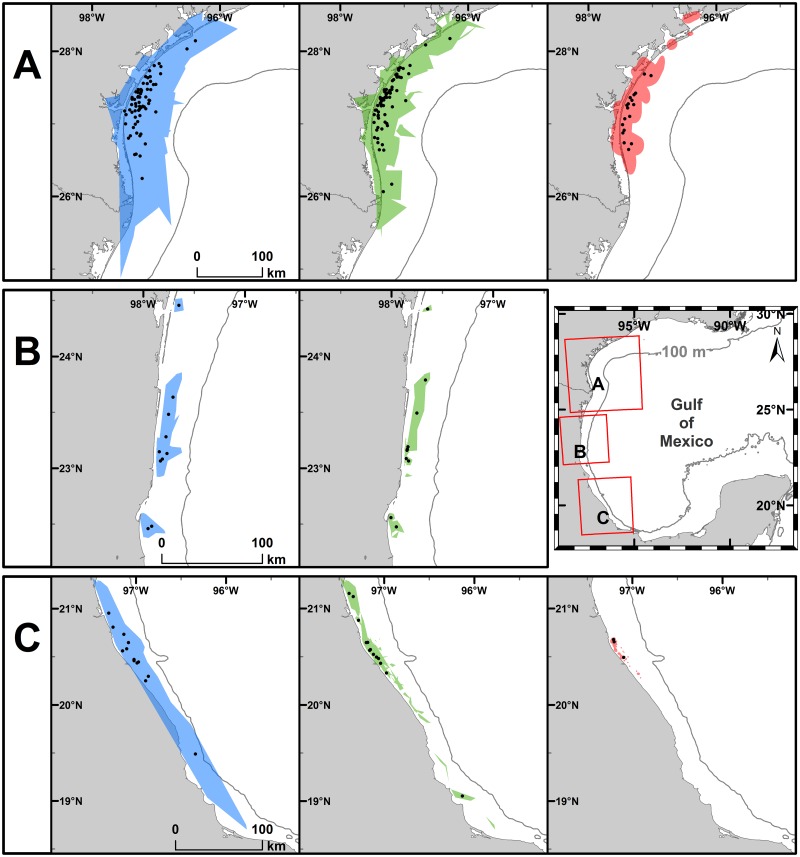
Home range [Minimum Convex Polygon (MCP) and α-Hull] and core use area [(Kernel Density Estimate (KDE)]. MCP (blue), α-Hull (green), and 50% KDE (red), with all centroids (•) in inter-nesting habitat for Kemp’s ridley turtles tracked after nesting at (A) Padre Island National Seashore, Texas, USA during 1998 through 2013 (n = 58), (B) Rancho Nuevo, Tamaulipas, Mexico during 2010 and 2011 (n = 9), and (C) Tecolutla, Veracruz, Mexico during 2012 and 2013 (n = 11). Note that it was not possible to estimate KDEs for RN since a cutoff of at least 20 days with mean daily locations was used and no turtles met that criterion.

We obtained 1272 total mean daily locations for the 82 tracks (Fig 2). Twenty-eight of the tracks (n = 30 centroids) were judged to exhibit site fidelity when p > 0.95 (S3 and S4 Tables). For the 78 inter-nesting tracks (92 inter-nesting periods) with five or more locations, we estimated home range using MCP and α-Hull methods (Fig 3). Additionally, for 21 of the 78 tracks with longer inter-nesting periods (i.e., where mean daily locations could be calculated for ≥ 20 days), we also calculated core use (Fig 3) and home ranges using KDE; it was not possible to calculate KDE for any of the RN tracks since none had ≥ 20 mean daily locations during inter-nesting periods.

Home range area estimated by MCP (mean ± SD = 1878.2 ± 2279.5 km^2^, range = 3.4 to 9413.2 km^2^, n = 92) was intermediate between that estimated by 95% KDE (mean ± SD = 2948.2 ± 2690.6 km^2^, range = 200.2 to 8609.2 km^2^, n = 21) and α-Hull (mean ± SD = 725.0 ± 1034.6 km^2^, range = 2.5 to 4667.4 km^2^, n = 92) (Table 1). MCP area for SSM turtles ranged from 3.4 to 7149.8 km^2^ (mean ± SD = 800.7 ± 1154.3 km^2^, n = 56) and for non-SSM turtles ranged from 42.3 to 9413.2 km^2^ (mean ± SD = 3554.4 ± 2582.4 km^2^, n = 36). Median MCP area was larger for non-SSM turtles than for SSM turtles (Mann-Whitney U Statistic = 290.000, T = 2392.000, n(non-SSM) = 36, n(SSM) = 56, p < 0.001). Median MCP area was larger for SSM tracks from VC than from RN (Dunn’s Method All Pairwise Multiple Comparison Procedure, p < 0.050), but did not significantly differ for other pairwise comparisons by nesting beach (p > 0.050).

### Distance to shore

Distance to shore was a significant predictor of turtle-days spent in distance categories during inter-nesting (Chi-square χ^2^ = 898.268 df = 6, p < 0.001); turtles spent longer periods in grid cells closer to shore. Distance to nearest land from MCP centroids (mean ± SD = 11.4 ± 8.1 km, range = 0.4 to 42.7 km, n = 92) was larger than distance to nearest land from 50% KDE centroids (mean ± SD = 6.0 ± 3.9 km, range = 1.7 to 14.9 km, n = 21) and distance to nearest land from α-Hull centroids (mean ± SD = 7.3 ± 6.2 km, range = 0.5 to 37.6 km, n = 92) (Table 1). The proportion of centroids in distance from shore categories varied for SSM versus non-SSM turtles (Chi-square χ^2^ = 71.390, df = 6, p < 0.001). MCP centroid distance to shore for SSM turtles ranged from 0.4 to 25.8 (mean ± SD = 7.6 ± 5.2 km, n = 56) and for non-SSM turtles ranged from 3.5 to 42.7 (mean ± SD = 17.1 ± 8.4 km, n = 36). Median MCP centroid distance to shore was larger for non-SSM turtles than for SSM turtles (Mann-Whitney U Statistic = 331.000, T = 2351.000, n(non-SSM) = 36, n(SSM) = 56, p < 0.001). Size of turtles and mean distance to shore values per MCP centroid were significantly correlated (Pearson Product Moment Correlation r = -0.234, p = 0.0248, n = 92). Median MCP centroid distance to land for SSM tracks did not differ by nesting beach (Kruskal-Wallis One Way ANOVA on ranks H = 4.504, df = 2, p = 0.105).

### Depth

**Fig 4:**
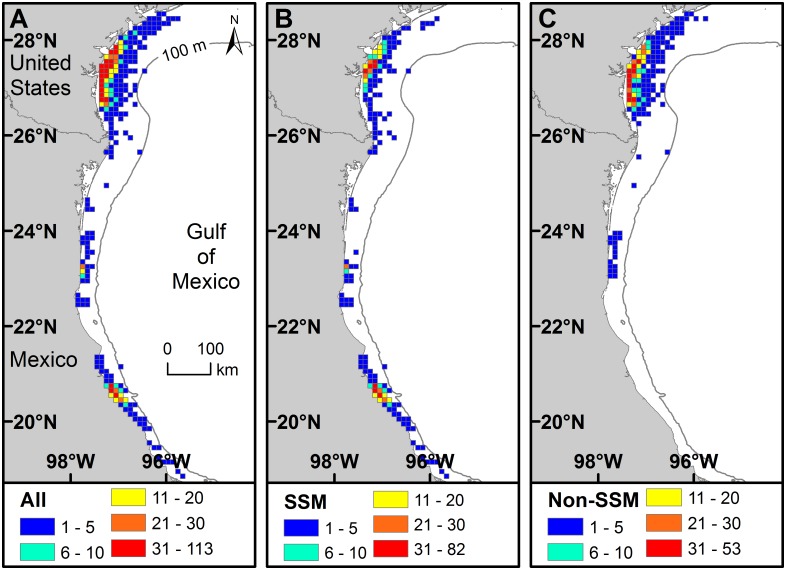
Number of Kemp’s ridley turtle-days spent for (A) all turtles, (B) State-Space Modeled (SSM) turtles, and (C) non-SSM turtles. Each panel includes the number of days each turtle was observed (turtle-days) in each grid cell using all Argos-retrieved filtered locations during the inter-nesting period except LC Z.

**Fig 5:**
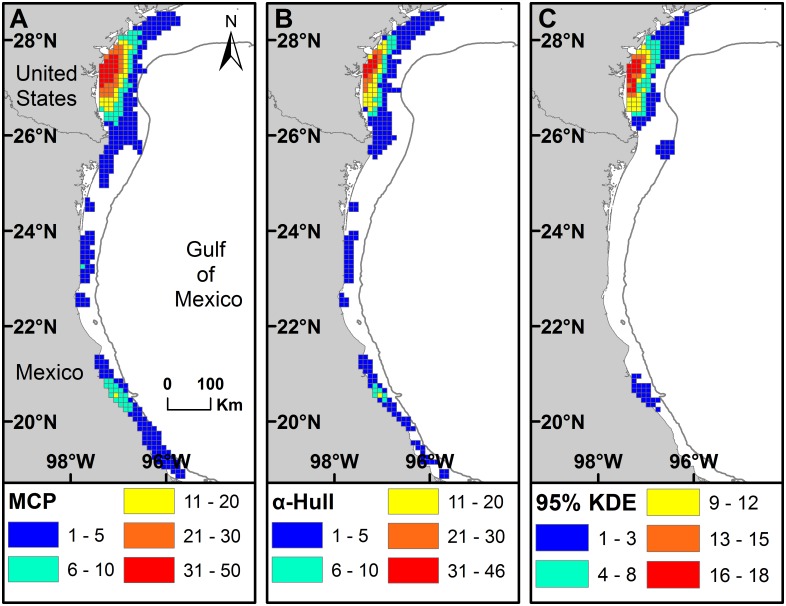
The number of individual Kemp’s ridley turtles with (A) Minimum Convex Polygons (MCPs), (B) α-Hull, and (C) Kernel Density Estimates (KDEs: 95%) for home range in each 10 km grid cell. Each inter-nesting period was counted once throughout all grid cells in each of the A, B, and C figures.

**Fig 6:**
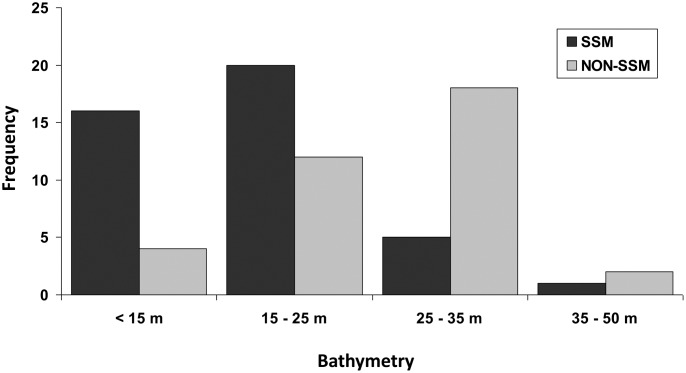
Bathymetry histogram for inter-nesting habitat Minimum Convex Polygon (MCPs) depth centroid locations for Kemp’s ridley turtles. Includes n = 58 tracked after nesting at Padre Island National Seashore, Texas, USA during 1998 through 2013, n = 9at Rancho Nuevo, Tamaulipas, Mexico during 2010 and 2011, and n = 11 at Tecolutla, Veracruz, Mexico during 2012 and 2013. SSM = depth centroids for turtles for which state space modeling was successful (n = 56); NON-SSM = depth centroids for turtles for which SSM was not possible (n = 36).

High numbers of turtle-days (Fig 4) and numbers of turtles (Fig 5) per 10 x 10 km grid cell occurred during inter-nesting in locations adjacent to nesting beaches. Bathymetry was a significant predictor of turtle-days spent in depth categories during inter-nesting (Chi-square χ^2^ = 1010.902, df = 4, p < 0.001); turtles spent longer periods in shallower grid cells. Bathymetry values (i.e., a proxy for water depths at centroid locations) for MCP (mean ± SD = 19.4 ± 9.2 m, range = 0 to 50 m, n = 92) were larger than for 50% KDE (mean ± SD = 14.3 ± 6.3 m, range = 2 to 27 m, n = 21) and α-Hull (mean ± SD = 14.8 ± 7.9 m, range = 1 to 40 m, n = 92). The proportion of centroids in depth categories varied for SSM versus non-SSM turtles (Chi-square χ^2^ = 24.254, df = 3, p < 0.001) (Fig 6). MCP centroid depth for SSM turtles ranged from 0 to 50 m (mean ± SD = 16.0 ± 8.9 m, n = 56) and for non-SSM turtles ranged from 9 to 45 m (mean ± SD = 24.7 ± 6.9 m, n = 36). Median MCP centroid depth was deeper for non-SSM turtles than for SSM turtles (Mann-Whitney U Statistic = 380.500, T = 1046.500, n(non-SSM) = 36, n(SSM) = 56, p < 0.001). Size of turtles and mean bathymetry values per MCP centroid were not significantly correlated (Pearson Product Moment Correlation r = -0.110, p = 0.295, n = 92). Median MCP centroid depths for SSM tracks did not differ by nesting beach (Kruskal-Wallis One Way ANOVA on ranks H = 2.375, df = 2, p = 0.305).

### Distance moved

**Fig 7:**
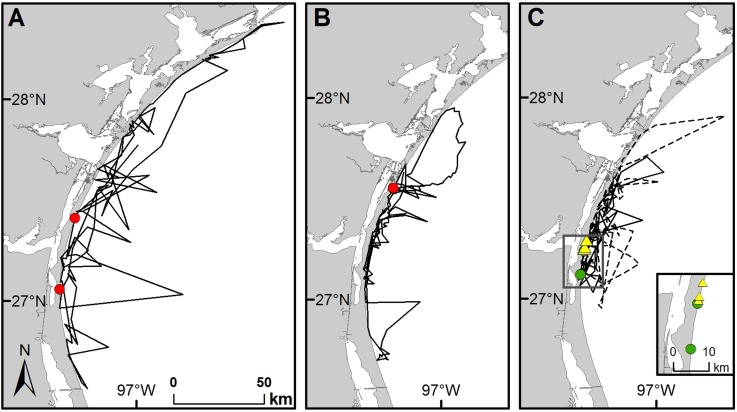
Examples of three different types of behavior for Kemp’s ridley turtles during the inter-nesting period. (A) No fidelity: Turtle P42, Tag number 17804 (non-SSM) did not display nest site fidelity between two confirmed nests (red circles, 36.8 km apart) or site fidelity to any in-water habitat during the 2003 nesting season. (B) Nest site fidelity only: Turtle P28, Tag number 82214 (non-SSM) displayed nest site fidelity between two confirmed nests (red circles, 0.3 km apart) but not site-fidelity to any in-water habitat during the 2008 nesting season. (C) Nest site and in-water fidelity: P121 displayed both nest-site and in-water habitat fidelity between two confirmed nests (green circles, 12.9 km apart) during the 2007 nesting season (Tag number 70703 (non-SSM), solid line) and three confirmed nests (yellow triangles, 1.1 and 4.0 km apart) during the 2012 nesting season (Tag number 112758 (SSM), dashed line).

TDM in-water during the inter-nesting period ranged from 9.6 to 1408.4 km (mean ± SD = 398.4 ± 327.9 km). The daily mean distance moved (TDM divided by number of days in the inter-nesting period), ranged from 4.8 to 38.1 km/day (mean ± SD = 17.5 ± 6.4 km/day, n = 81). SSM turtles traveled from 4.8 to 38.1 km/day (mean ± SD = 15.6 ± 6.5 km/day, n = 43) and non-SSM turtles traveled from 11.1 to 37.7 km/day (mean ± SD = 19.7 ± 5.6 km/day, n = 38). TDM for non-SSM tracks was greater than for SSM tracks (Mann-Whitney U Statistic = 518.500, T = 1856.500, n(non-SSM) = 38, n(SSM) = 43, p = 0.005). Movement behaviors during inter-nesting varied by turtle; while all remained in neritic habitat, some stayed relatively close to their previous nest site yet others ventured further in-water and/or had more distant subsequent nest sites (Fig 7).

### Potential overlap with anthropogenic activities

Inter-nesting in-water habitat overlapped with trawled areas (Fig 2). Of 155 centroid locations representing in-water inter-nesting habitat in U.S. GOM waters, 58% (n = 90) occurred in areas with 13,021–21,644 days of reported trawling effort from May through August of 2010–2013 and 42% (n = 65) occurred in areas with 21,645–45,167 days. One centroid was located in an inshore area, but this turtle inhabited very shallow GOM waters and had erroneous location reports inshore, resulting in a centroid location on the bay side of the barrier island (North Padre Island). Additionally, 7% of centroids in the U.S. and 43% of centroids in Mexico were within 15 km of oil and gas platforms.

## Discussion

Our results demonstrate that nearshore waters of the western GOM are vital inter-nesting habitat for Kemp’s ridley turtles. Although inter-nesting habitat has been defined for loggerhead (*Caretta caretta*), green (*Chelonia mydas*), hawksbill (*Eretmochelys imbricata*), leatherback (*Dermochelys coriacea*), flatback (*Natator depressus*) and olive ridley (*Lepidochelys olivacea*) turtles at various locales [26,28,29,31–33,51,57,77–79], this study provides the first quantification of inter-nesting habitat use by Kemp’s ridley turtles tracked after nesting at multiple beaches spanning most of the species’ nesting range. Inter-nesting sites overlapped with areas of shrimp trawling and oil and gas extraction activities, and our findings can be used to help develop strategies to protect Kemp’s ridley turtles from these activities and other threats. Satellite tracking data have been used to identify important inter-nesting, foraging, and migratory habitat of other sea turtle species, assess threats to sea turtles within those habitats, and aid with spatial planning for marine conservation in the USA [51,78,80,81] and other countries [30,34,82–88].

### Depth and distance from land

Inter-nesting habitat for Kemp’s ridley turtles is concentrated in relatively shallow, nearshore waters of the western GOM adjacent to, and immediately north and south of their nesting beaches. In-water, inter-nesting sites for Kemp’s ridley turtles in the GOM, delineated using MCP, were located a mean of 11.4 km from land and in a mean water depth of 19.4 m. When delineated by 50% KDE, inter-nesting sites were located a mean of 6.0 km from land and in a mean water depth of 14.3 m, and when delineated by α-Hull, inter-nesting sites were located a mean of 7.3 km from land and in a mean water depth of 14.8 m. Adult female Kemp’s ridley turtle foraging and migratory habitat delineated in the GOM using SSM [19,20] were further from land (mean = 25–26 km) but at relatively similar depth (mean = 18.5–20.0 m) compared to our estimates for inter-nesting sites. The location of inter-nesting habitat closer to shore than migratory and foraging habitat may be related to the contour of the GOM; important foraging and migratory corridor areas exist off the northern GOM, west coast of Florida, and Yucatan where the continental shelf extends much further from shore than in the western GOM off the nesting beaches in south Texas, Tamaulipas, and Veracruz.

In comparison, in-water, inter-nesting sites for loggerhead turtles in the northern Gulf delineated using 50% KDE were located a mean distance of 33.0 km from land, in water with a mean depth of 31.6 m, and delineated using MCP were located a mean 13.8 km from land, in water with a mean depth of 15.8 m [51]. Although the depth and distance delineated using MCP were very similar for those loggerheads and our Kemp’s ridley turtles, the distance from shore and depth delineated using 50% KDE were much larger for the loggerheads.

### Movements and home ranges

Kemp’s ridley turtles were mobile in nearshore GOM waters, and traveled a mean total distance of 399 km during the inter-nesting period. This is less than the mean total distance that loggerhead sea turtles traveled within inter-nesting habitat in the northern GOM (1422 km; [51]), but loggerheads may remain in inter-nesting habitat longer because they lay more clutches during a season [89]. Corrected for time, mean distances traveled during the inter-nesting period were similar for Kemp’s ridleys (17.5 km/day) and loggerheads (22.8 km/day; [51]). Kemp’s ridleys occupied relatively large home ranges during the inter-nesting period, and our mean MCP estimate for SSM Kemp’s ridley turtles (800.7 km^2^) were also similar to the mean MCP estimate for SSM loggerheads in the northern GOM (741.4 km^2^; [51]). Our mean 50% KDE estimate for SSM and non-SSM turtles collectively (895.3 km^2^, n = 21) was similar to the mean 50% KDE estimate for Kemp’s ridley turtles on the upper Texas coast (971^2^ km, n = 5) [23], but both were much larger than for 50% KDE found for loggerheads with successful SSM runs (61.9 km^2^, n = 10) [51]. Some turtles that we tracked exhibited site fidelity to an in-water, inter-nesting location. However, others failed the site fidelity test and showed no loyalty to a particular inter-nesting location, which may indicate plasticity in Kemp’s ridley turtle behavior during the inter-nesting period, as has been noted for some other sea turtle species [27,51,79,90–92].

We used KDE, MCP, and α-Hull to identify home range and core use areas since all three methods have been used in previous sea turtle studies. This allows comparison both across studies and methodologies. However, each method has its own limitations that must be considered. An MCP estimate most likely over-estimates the home range as it gives equal value to all points and clustering of points has no impact. The α-Hull estimates a smaller home range using an alpha value and triangulation to identify and remove outlying points; this reduces stochastic effects, likely providing a more accurate estimate of home ranges than the MCP. KDE is a non-parametric function which gives weight to each data point and produces an estimate of the most likely area where an animal will be found. The probability of encounter can be chosen; we chose a relatively low probability for home range representing a 50% chance of the animal being within the area. This method was the most conservative home range estimator used, and removed distant location points from consideration. However, we had fewer estimates of KDE than of MCP and α-Hull because we were unable to estimate KDE for several of the tracks, particularly those of shorter duration. Despite these differences in computational procedures, our mean MCP estimate for SSM Kemp’s ridley turtles (800.7 km^2^), mean 50% KDE estimate for SSM and non-SSM turtles collectively (895.3 km^2^), and mean α-Hull estimate for SSM and non-SSM turtles collectively (725.0 km^2^) were relatively similar, but the mean MCP estimate for SSM and non-SSM turtles combined (1878.2 km^2^) was much larger.

We included results from both SSM and non-SSM turtles, to maintain as much data as possible for our analyses. KDE, MCP, and α-Hull estimates for mean area, depth, and distance from shore were all less for turtles with successful SSM runs than for those with unsuccessful SSM runs. The standard deviations of most of these mean values were also less for the successful SSM runs than the unsuccessful runs. We recommend that future analyses of home range and core use areas utilize SSM-derived values whenever possible.

Kemp’s ridleys may be moving during the inter-nesting period to forage. During the inter-nesting period, female sea turtles develop the next clutch of eggs to be laid [93]. Egg production is energetically demanding, and strategies used by sea turtles for energy optimization during the inter-nesting period appear to vary and be related to food availability [32,90,94]. Turtles do not eat during the inter-nesting period in areas where food is unavailable, and minimize energy loss by spending most of their time resting and undertaking more restricted movements [27,32,90,95]. In contrast, where food is available, sea turtles sometimes forage during the inter-nesting period to augment energy reserves, but they undertake more extensive movements and occupy comparatively larger home ranges to do so [57,90,94,96,97]. In the western GOM, adult Kemp’s ridleys forage primarily on Portunid and other crab species [98,99]. Less is known about habitat use of adult male Kemp’s ridleys, but the majority of males tracked near RN and PAIS to date remained in the vicinity of those areas year-round [18][Shaver, unpublished data]. These data indicate ample foraging resources are available there for resident males to exploit, which adult females could potentially consume during the inter-nesting period. Whether Kemp’s ridleys forage during the inter-nesting period remains to be tested, but animal-borne digital cameras, time depth recorders, accelerometers, or three-dimensional data loggers [100–106] may be useful for such investigation.

During the inter-nesting period, Kemp’s ridley females may also move to spread nests within the region, mate or avoid males, avoid predators, or seek other conditions (e.g., warmer or calmer waters, less disturbance, etc.) before nesting again, as has been suggested for some other sea turtles [27,32,33]. We recorded 1.4 nests per female from the time of PTT deployment through the end of the inter-nesting period, but some other nests may have been missed during this time. Although some researchers have identified sea turtle nests from satellite tracking data [107], this was not possible from our data set due to limited precision of Argos location data, duty cycles of less than 24 h d^-1^ (i.e., 6/h on/6 h off) for many of the PTTs, and quick nesting habits of the species (see [21]). In contrast, kinship analysis has been used to assign nests of unknown maternity to nesting Kemp’s ridleys in Texas [74]; the 6 nests assigned to turtles in this study were within the tracking range documented for these turtles during the inter-nesting period. Nest site fidelity was not correlated with in-water distance moved during the inter-nesting period, and nesting habits of the species may have affected both. Unlike other sea turtles, ridleys (*Lepidochelys spp*.) often nest in synchronous emergences called arribadas, although some solitary nesting also occurs in both species. The interval between nests is more variable and influenced by environmental cues that trigger arribada formation [15,99,108]. Kemp’s ridley turtle arribadas tend to occur on windy days and may be initiated by increases in wind speed and surf which in turn trigger auditory, olfactory, or visual cues that stimulate mass nesting [15,99,108–111]. Although they produce eggs as rapidly as other sea turtle species, a variable inter-nesting interval enables Kemp’s ridley females to delay egg deposition until environmental cues initiate an arribada [108,112]. In Tamaulipas, arribadas occur more or less simultaneously along 100 km or more of beach [108]. For females that we detected nesting more than once after PTT deployment at PAIS, we found a mean distance between nests of 18.7 km (range = 0.3 to 77.3 km). About 95% of Kemp’s ridleys documented nesting more than once on the Texas coast between 1991 and 2014 nested on the same or nearby beaches, but occasionally some individuals nested on widely separated beaches within a year or during different years [8]. However, because these beaches can be quite long (up to 130 km), only a fraction of the 95% that nested on the same or nearby beaches exhibited site fidelity as defined here (13.5 km or less between nest sites). Low nest site fidelity could be due to the smaller numbers of nesting turtles and less well-developed arribadas at PAIS compared to RN [8]. Low nest site fidelity could also be due to the inter-nesting habits of the species. During the inter-nesting period, some Kemp’s ridleys transit up to 200 km away from their previous nest site [21,24]. When appropriate environmental conditions arise for nesting, perhaps in the presence of other turtles, Kemp’s ridley females may come ashore to nest where they are even if it is at a different location than where they nested previously.

During the inter-nesting period, Kemp’s ridley females may have engaged in mating. Sea turtles are known to mate in between nesting events [113], and Kemp’s ridley turtle [114] and other sea turtle species have been shown to exhibit multiple paternity within their clutches (see [115]). However, Kemp’s ridley females may have also moved to waters away from their previous nest site to avoid males seeking to mate. Sea turtles are promiscuous and males often engage in aggressive courtship behavior to gain access to females [115,116]. Movements of females to avoid males have been noted in some species [100] and Rostal [117] observed adult female Kemp’s ridleys in captivity attempting to avoid mating by using refusal behavior. Unmanned aerial vehicle (UAV) technology [118] may be effective to study these behaviours during the inter-nesting period for turtles marked after nesting using a means visible aerially.

### Inter-nesting periods and dates

Turtles occupy inter-nesting habitat during the time between their first and last clutches of the season. Overall inter-nesting season for individuals (from capture date to last inter-nesting point regardless of migration periods) in our study totaled 1838 days and ranged from 2 to 106 days (mean = 22.4 days), but they underestimate time in residence within inter-nesting habitat since not all PTTs were deployed on turtles immediately after their first clutch of the season. Additionally, most females likely remain offshore from the nesting beaches longer than the time between the first and last nests. Adult females enter the waters off the nesting beach in advance of their first clutch of the season, but less is known about the arrival times than their departure times from this habitat. Only three adult Kemp’s ridley females have been tracked from their foraging grounds to their nesting beaches. The first was tracked from the northern GOM, arrived off the RN nesting beach on 10 March 1995, moved an additional 100 km to the south, and then returned to nest at Rancho Nuevo on 23 April 1995 and 19 May 1995 [119]. The second was tracked from the northern GOM and arrived offshore from the PAIS nesting beach in late-March 2007 [20]. The third was tracked from the Yucatan, arrived offshore from the Tecolutla nesting beach around 11 April 2014, and was first observed nesting near Tecolutla that year on 30 April [20]. Tracking of adult females from the foraging grounds to the nesting beaches is needed to gain critical information about the timing of arrival of adult females to waters off the nesting beach. Most adult female Kemp’s ridley turtles transit to the foraging grounds after nesting is complete for the season, but a few of those tracked have remained off the nesting beach into the fall [21]. Shaver et al. [20] found that migration for post-nesting Kemp’s ridleys occurred from late-May through August. But, for all years combined, by 1 June, approximately half of all SSM turtles tracked were in migration mode (i.e., transiting from the nesting grounds to foraging sites) and by 1 August that proportion dropped to about 30%.

### Importance of inter-nesting habitat

The western GOM is vital habitat for the survival of this species. Virtually all nesting by this species occurs along western GOM shorelines, and thus, this region provides critical breeding, inter-nesting, migratory, and foraging habitat for adult females. Less is known about the habits of males, but based on satellite tracking conducted to-date it appears that most adult males remain in the western GOM year-round [18]. In-water, inter-nesting habitat overlaps with trawling, oil and gas extraction, and other human related threats. Although shrimp trawling effort has declined off the Texas coast in recent years [120,121], and is prohibited in GOM waters off south Texas out to 8 km from shore from 1 December through 15 May [122] and off the entire Texas coast out to 370 km from 15 May through 15 July, these waters are open to shrimp trawling for the remainder of the year. A reserve protects Kemp’s ridleys off the RN nesting beach, but it does not encompass waters off the entire nesting epicenter in Tamaulipas. Results from this study could be used to adjust the temporal and spatial boundaries of these closures and reserves to enhance protection of turtles there. For example, based on the mean distance to shore of 6–17 km as estimated by 50% KDE, MCP, and α-Hull methodologies for turtles tracked from PAIS, it may be advisable to extend the seasonal closure further offshore than the current 8 km. Our results could also be used to establish and delineate boundaries for a similar in-water protection zone for Kemp’s ridley turtles off the primary nesting beaches in Mexico. Additionally, our results could be used to help establish other protection measures to mitigate for threats that may emerge in the future. Since nearly all reproduction by this species occurs in the western GOM, success of Kemp’s ridley turtle recovery efforts depends upon bi-national efforts to protect adults on and offshore from these vital nesting beaches.

## Supporting information

S1 Table Platform Transmitter Terminals (PTTs) specifications including type and duty cycle of PTTs used, and accuracy of Argos Location Classes for nesting Kemp’s ridley turtles (*Lepidochelys kempii*) tracked in the western Gulf of Mexico. (PDF)

S2 Table Summary of satellite-tracking details for nesting Kemp’s ridley turtles in the western Gulf of Mexico. (PDF)

S3 Table Kernel Density Estimation (KDE), Minimum Convex Polygon (MCP), and α-Hull results for nesting Kemp’s ridley turtles with successful State-Space Model (SSM) runs. (PDF)

S4 Table Kernel Density Estimation (KDE), Minimum Convex Polygon (MCP), and α-Hull results for nesting Kemp’s ridley turtles with unsuccessful State-Space Model (SSM) runs. (PDF)

S1 Fig Straight carapace length of n = 82 Kemp’s ridley turtles tracked by satellite telemetry after nesting on beaches in the western Gulf of Mexico. (TIF)

S2 Fig Mean distance between nests for n = 28 Kemp’s ridley turtles tracked by satellite telemetry after nesting at Padre Island National Seashore, Texas and documented nesting at least one more time during that nesting season. (TIF)
