# Supplementary material for: Inter-nesting movements and habitat-use of adult female Kemp’s ridley turtles in the Gulf of Mexico
Source: PLoS One. 2017 Mar 20;12(3):e0174248. doi: 10.1371/journal.pone.0174248 (PMC5358874; doi:10.1371/journal.pone.0174248)
Supplement: S1 Table — (PDF) [file pone.0174248.s001.pdf]

S1 Table. Platform transmitter terminals (PTTs) specifications including type and duty cycle of PTTs used, and accuracy of Argos Location Classes for nesting Kemp's ridley turtles (*Lepidochelys kempii*) tracked in the western Gulf of Mexico.

PTT Specifications
PTT Models
Telonics, Inc. (Mesa, Arizona, USA) ST-6, ST-18, ST-20 (n=37)
Sirtrack (Haverloc North, New Zealand) KS-101, KS-202 (n=20)
Wildlife Computers (Redmond, WA, USA) MK-10A, MK-AF, SPOT5 (n=25)
Duty Cycles by Year
6 h on/6 h off (1998 through 2007)
24 h d^{-1} for the first 106 days and then 6 h on/6 h off (2008)
6 h on/6 h off for KS-101, KS-202 (2010 through 2013)
24 h d^{-1} for MK-10A, MK10AF, SPOT5 (2010 through 2013)
Argos Assigned Accuracy Estimates of Location Classes
LC3: <250 m
LC2: 250-<500 m
LC1: 500-<1500 m
LC0: >1500 m
LCA: unknown
LCB: unknown
LCZ: unknown
