# Supplementary material for: Inter-nesting movements and habitat-use of adult female Kemp’s ridley turtles in the Gulf of Mexico
Source: PLoS One. 2017 Mar 20;12(3):e0174248. doi: 10.1371/journal.pone.0174248 (PMC5358874; doi:10.1371/journal.pone.0174248)
Supplement: S2 Table — (PDF) [file pone.0174248.s002.pdf]

S2 Table. **Summary of satellite-tracking details for nesting Kemp's ridley turtles (*Lepidochelys kempii*) in the western Gulf of Mexico.**

Turtle ID #	Tag no.[a]	SCL (cm)	Tracking duration (days)[b]	Inter-nesting period (days)[c]	No. inter-nesting mean daily locations	SSM (no. of cen-troids)[d]	No. nests (mean inter-nesting interval in days)	Mean distance (km) between recorded nests (no. of distances)	Total distance moved (TDM) during inter-nesting period (km)	TDM/day (km)
Padre Island National Seashore, Texas, USA										
P08†	7689	62.9	4/25/1998-8/31/1998(128)	4/30/1998-5/22/1998(18)*	10, 5	YES(2)	2(26)	5.1(1)	339.4	18.9
P09†	7681A	62.0	4/25/1998-8/31/1998(128)	5/8/1998-6/9/1998(33)	8	NO(1)	1	NA	535.6	16.2
P11	21811	61.0	5/22/1998-8/31/1998(101)	5/26/1998-6/12/1998(18)	7	NO(1)	1	NA	199.3	11.1
P14	18281	64.1	4/9/1999-8/31/1999(144)	4/13/1999-6/29/1999(78)	34	NO(1)	2(20)	1.5(1)	1323.3	17.0
P16	18160	62.3	5/6/1999-8/31/1999(117)	5/7/1999-5/21/1999(15)	6	NO(1)	1	NA	234.2	15.6
P18	18277	62.2	5/10/1999-5/27/1999(17)	5/11/1999-5/16/1999(6)	5	NO(1)	1	NA	164.9	27.5
P15†	18308	59.9	4/20/1999-8/31/1999(133)	4/21/1999-6/29/1999(70)	37	NO(1)	2(35)	5.7(1)	1408.4	20.1
P19	18299	62.2	5/26/1999-8/31/1999(97)	5/31/1999-6/2/1999(3)	2	NO(0)	1	NA	57.8	19.3
P20†	18301	61.6	5/26/1999-8/31/1999(97)	5/28/1999-6/3/1999(7)	4	NO(0)	1	NA	95.6	13.7
P21†	18277A	60.8	4/19/2000-7/25/2000(97)	4/20/2000-5/13/2000(24)	20	NO(1)	1	NA	578.5	24.1
P22	21811A	63.1	4/13/2000-8/31/2000(140)	4/13/2000-4/15/2000(3)	3	YES(1)	1	NA	16.2	5.4
P23	24857	63.5	4/22/2000-8/31/2000(131)	4/23/2000-5/17/2000(25)	15	NO(1)	1	NA	432.0	17.3
P08†	29351	63.0	4/24/2001-8/31/2001(129)	4/27/2001-5/11/2001(15)	11	NO(1)	1	NA	274.6	18.3
P20†	24858	61.9	5/16/2001-8/31/2001(107)	5/18/2001-6/16/2001(30)	18	NO(1)	1	NA	586.3	19.5
P28†	29350	63.1	5/20/2001-8/31/2001(103)	5/21/2001-6/20/2001(31)	17	NO(1)	1	NA	533.9	17.2
P30†	25220	61.2	4/24/2002-8/31/2002(129)	4/25/2002-5/31/2002(37)	28	NO(1)	1	NA	855.8	23.1
P12†	25217	60.9	4/26/2002-8/31/2002(127)	4/27/2002-5/27/2002(31)	28	NO(1)	2(18)	26.6(1)	826.9	26.7
P31	25218	64.7	4/27/2002-8/31/2002(126)	4/28/2002-5/16/2002(19)	17	NO(1)	1	NA	716.5	37.7
P21†	25219	61.5	4/29/2002-8/31/2002(124)	4/30/2002-5/18/2002(19)	13	NO(1)	1	NA	417.1	22.0
P45	15520	61.7	5/13/2003-8/31/2003(110)	5/14/2003-5/29/2003(16)	7	NO(1)	1	NA	283.1	17.7
P42	17804	60.1	4/9/2003-8/31/2003(144)	4/10/2003-5/31/2003(52)	44	NO(1)	2(17)	36.8(1)	1161.3	22.3
P43	17806	61.4	5/5/2003-8/31/2003(118)	5/6/2003-5/9/2003(4)	4	NO(1)	1	NA	80.3	20.1

P44	17807	66.5	5/6/2003-8/31/2003(117)	5/7/2003-5/18/2003(12)	8	NO(1)	1	NA	234.9	19.6
P54	47789	64.2	5/1/2004-8/31/2004(122)	5/2/2004-5/12/2004(11)	8	YES(1)	2(25)	5.1(1)	91.0	8.3
P21†	47790	61.3	5/1/2004-8/31/2004(122)	5/2/2004-5/10/2004(9)	5	YES(1)	1	NA	186.7	20.7
P55	47791	63.3	5/2/2004-8/31/2004(121)	5/3/2004-6/6/2004(35)	25	NO(1)	2(22)	77.3(1)	520.3	14.9
P09†	53628	62.4	4/23/2005-5/1/2005(8)	4/24/2005-5/1/2005(8)	5	NO(1)	1	NA	126.8	15.8
P68†	53629	63.3	4/28/2005-8/31/2005(125)	4/29/2005-5/25/2005(27)	24	NO(1)	2(41)	3.4(1)	583.9	21.6
P69	53630	57.2	4/28/2005-8/31/2005(125)	5/15/2005-6/29/2005(46)	36	NO(1)	1	NA	898.0	19.5
P28†	53631	63.8	5/8/2005-8/31/2005(115)	5/8/2005-5/21/2005(14)	11	YES(1)	2(22)	7.8(1)	298.0	21.3
P30†	62822	62.1	4/27/2006-8/31/2006(126)	4/27/2006-5/8/2006(12)	12	YES(1)	3(25.5)	28.5(2)	259.5	21.6
P12†	62943	61.3	4/27/2006-8/31/2006(126)	4/27/2006-5/15/2006(19)	13	YES(1)	2(26)	20.3(1)	434.1	22.8
P84	62823	60.8	4/27/2006-8/3/2006(98)	4/27/2006-6/3/2006(38)	31	YES(1)	3(17.5)	6.2(2)	811.2	21.3
P109	70700	63.7	5/1/2007-8/31/2007(122)	5/1/2007-5/19/2007(16)*	4, 11	YES(2)	2(19)	9.1(1)	211.4	13.2
P35	70701	63.6	5/1/2007-8/31/2007(122)	5/2/2007-6/4/2007(34)	33	NO(1)	1	NA	756.7	22.3
P104	70702	60.6	5/1/2007-8/31/2007(122)	5/2/2007-6/29/2007(59)	50	NO(1)	2(19)	23.2(1)	1023.5	17.3
P121†	70703	62.9	5/2/2007-8/31/2007(121)	5/3/2007-6/7/2007(36)	27	NO(1)	2(37)	13.0(1)	500.3	13.9
P28†	82214	64.2	4/24/2008-7/31/2008(98)	4/25/2008-5/22/2008(28)	28	NO(1)	2(28)	0.3(1)	878.2	31.4
P33	82215	67.0	4/24/2008-8/31/2008(129)	5/5/2008-6/4/2008(18)*	3, 14	YES(2)	1	NA	689.7	38.1
P92	82216	65.5	4/24/2008-8/31/2008(129)	4/25/2008-5/19/2008(25)	25	NO(1)	1	NA	786.6	31.5
P120	47519	64.1	4/27/2010-8/29/2010(124)	5/2/2010-5/15/2010(14)	13	YES(1)	1	NA	277.0	19.8
P15†	47529	60.7	5/6/2010-8/31/2010(117)	5/7/2010-6/29/2010(54)	45	NO(1)	2(18)	14.0(1)	1082.1	20.0
P125	47562	63.6	5/6/2010-8/31/2010(117)	5/6/2010-5/25/2010(20)	20	YES(1)	2(18)	9.0(1)	321.5	16.1
P298	47690	60.5	5/11/2010-8/6/2010(87)	5/11/2010-5/31/2010(21)	19	YES(1)	2(29)	15.9(1)	450.1	21.4
P315	47709	61.8	6/9/2010-8/31/2010(83)	6/11/2010-6/17/2010(7)	5	NO(1)	1	NA	97.0	13.9
P319	101136	59.8	4/7/2011-8/31/2011(146)	4/7/2011-4/21/2011(11)*	8, 2	YES(2)	1	NA	205.1	18.6
P164	101137	63.7	4/16/2011-8/31/2011(137)	4/18/2011-5/7/2011(13)*	2, 8, 2, 1	YES(4)	2(22)	24.7(1)	202.4	15.6
P321	101138	62.3	4/22/2011-8/31/2011(131)	4/22/2011-4/25/2011(4)	4	YES(1)	3(15.5)	19.7(2)	55.5	13.9
P172	101139	64.8	4/23/2011-8/31/2011(130)	4/23/2011-6/4/2011(31)*	11,12,1,5	YES(4)	3(22.5)	7.9(2)	544.2	17.6
P145	101140	64.5	4/23/2011-8/20/2011(119)	5/11/2011-5/22/2011(12)	11	YES(1)	2(25)	1.9(1)	233.9	19.5
P280	106339	62.8	4/23/2011-8/31/2011(130)	4/23/2011-5/18/2011(25)	10	NO(1)	2(25)	17.0(1)	436.4	16.8
P322	106341	61.7	4/25/2011-8/31/2011(128)	4/28/2011-5/24/2011(9)*	1, 2	YES(2)	2(23)	64.2(1)	63.5	7.1
P326	106346	60.3	4/28/2011-8/31/2011(125)	5/7/2011-5/18/2011(12)	4	YES(1)	2(20)	42.1(1)	217.6	18.1

P230	106347	61.4	4/28/2011-8/31/2011(125)	4/28/2011-5/3/2011(6)	3	YES(1)	2(41)	33.7(1)	144.2	24.0
P121†	112758	63.5	4/12/2012-8/31/2012(141)	4/19/2012-5/22/2012(34)	31	YES(1)	3(20.5)	2.6(2)	814.2	23.9
P113	117515	64.3	6/8/2012-8/20/2012(73)	6/8/2012-6/27/2012(20)	8	NO(1)	1	NA	327.2	16.4
P428	112766	62.7	5/25/2013-8/31/2013(98)	5/25/2013-6/4/2013(11)	11	YES(1)	1	NA	268.5	24.4
P431	117517	61.6	5/26/2013-8/31/2013(97)	5/26/2013-6/3/2013(9)	9	YES(1)	1	NA	102.1	11.3
P68†	117520	64.2	5/28/2013-8/31/2013(95)	5/28/2013-5/31/2013(4)	4	YES(1)	1	NA	67.9	17.0
P231	117521	61.7	5/28/2013-8/31/2013(95)	5/28/2013-6/1/2013(5)	5	NO(1)	1	NA	100.8	20.2
Rancho Nuevo, Tamaulipas, Mexico										
RN01	100396	66.4	7/21/2010-8/31/2010(41)	7/22/2010-7/25/2010(4)	1	NO(0)	1	NA	UN	UN
RN02	100397	63.3	7/21/2010-8/31/2010(41)	7/22/2010-7/25/2010(4)	2	NO(1)	1	NA	85.1	21.3
RN03	100398	63.5	7/23/2010-8/31/2010(39)	7/24/2010-8/3/2010(11)	5	NO(1)	1	NA	143.9	13.1
RN06	100391	65.8	4/28/2011-8/31/2011(125)	5/18/2011-6/8/2011(12)*	4, 2	YES(2)	2(78)	UN	149.1	12.4
RN07	100392	62.6	4/28/2011-8/31/2011(125)	5/6/2011-5/23/2011(18)	6	YES(1)	2(25)	UN	180.4	10.0
RN08	100393	62.0	4/28/2011-8/31/2011(125)	5/16/2011-6/5/2011(21)	7	YES(1)	1	NA	235.9	11.2
RN09	100394	63.5	4/28/2011-8/31/2011(125)	5/7/2011-6/6/2011(28)*	6, 3	YES(2)	1	NA	266.2	9.5
RN10	100395	65.2	4/28/2011-8/31/2011(125)	5/2/2011-6/7/2011(32)*	7, 4	YES(2)	1	NA	338.6	10.6
RN12	100403	63.6	6/5/2011-8/31/2011(87)	6/11/2011-6/29/2011(7)*	4, 3	YES(2)	1	NA	130.1	18.6
RN13	100404	67.0	6/5/2011-8/31/2011(87)	6/6/2011-6/7/2011(2)	2	YES(1)	1	NA	9.6	4.8
RN14	100405	65.2	6/5/2011-6/21/2011(16)	6/6/2011-6/7/2011(2)	2	NO(1)	1	NA	22.2	11.1
Techolutla, Veracruz, Mexico										
VC01	47530	62.0	5/20/2012-8/31/2012(103)	5/20/2012-9/2/2012(106)	99	YES(1)	1	NA	1116.6	10.5
VC02	101134	62.9	5/21/2012-7/12/2012(52)	5/21/2012-5/28/2012(8)	8	YES(1)	1	NA	159.6	19.9
VC03	101135	59.2	5/21/2012-8/31/2012(102)	5/21/2012-6/16/2012(27)	22	YES(1)	1	NA	187.8	7.0
VC04	126228	68.8	4/18/2013-7/19/2013(92)	4/18/2012-5/14/2013(27)	12	YES(1)	1	NA	185.6	6.9
VC05	126229	75.2	4/18/2013-7/18/2013(91)	4/21/2013-5/7/2013(17)	9	YES(1)	1	NA	236.7	13.9
VC06	126230	67.1	5/20/2013-8/12/2013(84)	5/20/2013-6/19/2013(31)	16	YES(1)	1	NA	403.5	13.0
VC09	126233	64.8	4/19/2013-8/31/2013(134)	4/20/2013-5/18/2013(29)	13	YES(1)	1	NA	413.8	14.3
VC10	126234	63.9	4/19/2013-8/31/2013(134)	4/20/2013-5/30/2013(41)	21	YES(1)	2(22)	UN	469.6	11.5
VC11	126235	62.8	4/19/2013-7/29/2013(101)	4/20/2013-5/8/2013(19)	10	YES(1)	1	NA	293.3	15.4
VC12	126236	65.2	4/19/2013-8/10/2013(113)	4/22/2013-5/27/2013(36)	16	YES(1)	1	NA	302.6	8.4
VC13	126237	67.7	5/21/2013-8/31/2013(102)	5/22/2013-6/23/2013(33)	15	YES(1)	1	NA	364.2	11.0

[a]All PTTs deployed at Padre Island National Seashore immediately after first recorded nest of the season except 117515 deployed after third recorded nest and 117521 deployed after second recorded nest; comparable data were not available for PTTs deployed in Mexico.

[b]Tracking duration = Either when transmitter stopped or until 31 August of tagging year (data analysis cut-off), whichever came first.

[c]Inter-nesting period = for SSM turtles as defined by SSM through the last "inter-nesting point", or for non-SSM turtles from the tagging date through last day before directional movement.

[d]YES = SSM turtle = track worked with SSM model; NO = non-SSM turtle = track did not work with SSM model; number of centroids = MCP and α-Hull centroids.

†This individual was repeatedly tracked after nesting at Padre Island National Seashore during different years.

*Contained multiple MCP and α-Hull centroids and multiple inter-nesting periods; inter-nesting period = from first day of first inter-nesting period to last day of last inter-nesting period; (days) = total number of days during the multiple inter-nesting periods; TDM/day = total distance moved during the multiple inter-nesting periods divided by the total number of days during the multiple inter-nesting periods.

NA = Not applicable.

UN = Unknown (sufficient data were not available to calculate).
