# Supplementary material for: Inter-nesting movements and habitat-use of adult female Kemp’s ridley turtles in the Gulf of Mexico
Source: PLoS One. 2017 Mar 20;12(3):e0174248. doi: 10.1371/journal.pone.0174248 (PMC5358874; doi:10.1371/journal.pone.0174248)
Supplement: S3 Table — (PDF) [file pone.0174248.s003.pdf]

S3 Table. **Kernel density estimation (KDE), minimum convex polygon (MCP), and α-Hull results for nesting Kemp's ridley turtles (*Lepidochelys kempii*) with successful state-space model (SSM) runs.**

		KDE					MCP			α-Hull		
Turtle ID #[a, b]	Tag no.	Band-width	50% area (km^2)[d]	50% centroid depth (m)	50% centroid to land (km)	95% area (km^2)[d]	Area (km^2)[d]	Centroid depth (m)	Centroid to land (km)	Area (km^2)[d]	Centroid depth (m)	Centroid to land (km)
Padre Island National Seashore, Texas, USA												
P08	7689						2176.4	20	13.9	1649.0	25	15.1
P08C2	7689						245.1	17	8.7	99.0	13	4.3
P22	21811A						3.4	1	0.8	3.4	1	0.8
P54	47789						405.3	18	8.5	220.9	17	8.1
P21	47790[c]						1222.6	22	13.5	520.7	17	7.6
P28	53631						2541.2	21	13.3	1106.4	14	5.3
P30	62822[c]						952.8	18	8.5	452.5	14	5.6
P12	62943						2121.3	25	17.8	706.4	17	6.7
P84	62823	0.2[e]	475.9	16	5.6	1603.2	2046.4	22	12.4	959.8	14	5.6
P109	70700						102.8	12	2.1	72.8	7	1.0
P109C2	70700						362.5	17	6.0	214.0	12	3.0
P33	82215						105.9	18	12.3	54.9	17	12.2
P33C2	82215[c]						1524.3	30	25.8	235.5	14	4.5
P120	47519[c]						829.0	14	7.2	224.2	9	1.2
P125	47562[c]	5216.0	281.0	15	6.6	956.4	565.4	19	9.6	268.9	17	6.8
P298	47690						7149.8	29	18.1	3158.1	24	13.3
P319	101136[c]						40.0	12	1.8	26.6	12	2.1
P319C2	101136						1031.1	19	8.9	281.1	11	2.6
P164	101137						261.3	11	3.8	101.2	10	2.4
P164C2	101137[c]						52.0	11	1.8	25.1	10	2.0
P164C3	101137						7.9	6	0.5	7.3	6	0.5
P321	101138						125.1	18	8.4	107.3	18	8.9
P172	101139						1019.7	16	7.1	531.9	14	4.7

P172C2	101139[c]						268.6	13	4.2	62.1	11	1.9
P172C3	101139[c]						5.5	0	0.4	2.5	7	0.5
P172C4	101139						270.4	14	4.0	90.1	12	2.1
P145	101140[c]						382.4	15	5.3	127.3	10	3.1
P322	106341						70.8	11	3.0	11.6	12	3.9
P322C2	106341						56.9	23	18.5	56.9	23	18.5
P326	106346						1803.3	20	10.8	165.3	6	1.5
P230	106347						731.2	19	8.5	180.5	17	7.0
P121	112758[c]	3061.6	277.3	11	3.0	1258.5	1987.6	20	11.2	487.0	14	4.2
P428	112766						861.0	20	10.9	246.2	19	9.2
P431	117517[c]						151.8	19	9.3	44.4	19	9.5
P68	117520						136.0	13	5.1	28.4	19	9.8
Rancho Nuevo, Tamaulipas, Mexico												
RN06	100391						115.4	0	1.2	58.3	2	2.5
RN06C2	100391						27.0	3	2.1	17.1	2	1.5
RN07	100392						336.9	15	5.3	59.5	6	2.0
RN08	100393[c]						112.0	7	2.0	30.8	6	1.0
RN09	100394[c]						206.9	13	5.1	142.4	12	5.1
RN09C2	100394[c]						252.7	20	8.1	25.3	6	2.6
RN10	100395						253.5	21	8.5	39.5	6	0.5
RN10C2	100395[c]						314.6	31	9.8	23.4	7	1.6
RN12	100403[c]						97.9	8	2.9	36.6	18	4.4
RN12C2	100403						119.3	6	1.8	53.5	11	3.0
Tecolutla, Veracruz, Mexico												
VC01	47530[c]	982.5	44.5	2	2.7	298.4	1127.3	12	13.2	154.4	3	4.3
VC02	101134						509.5	2	3.6	345.0	5	3.6
VC03	101135	1332.9	44.7	4	2.7	200.2	661.4	10	5.8	74.8	2	2.6
VC04	126228[c]						212.4	23	5.8	20.4	6	1.3
VC05	126229						219.2	8	1.3	65.1	6	0.9
VC06	126230						3522.0	50	12.2	345.5	20	9.0
VC09	126233						1259.7	26	7.3	184.0	2	2.0

VC10	126234[c]	1148.3	49.0	2	1.7	210.2	474.9	8	4.7	96.8	4	2.8
VC11	126235						1610.6	13	10.4	569.8	5	6.9
VC12	126236						610.8	6	7.2	129.1	3	2.7
VC13	126237						1177.9	29	7.8	128.0	4	3.6

[a]C2 = Centroid 2 for a track, C3 = Centroid 3 for a track, and C4 = Centroid 4 for a track.

[b]Does not include Tag no. 100404 which had a successful SSM run and passed the site fidelity test. Although this had distance, home ranges could not be calculated so it was excluded from home range maps and this table.

[c]p values for site fidelity test were > 0.95.

[d]These values include only in-water area; any land within KDE, MCP, and α-Hull contour was removed from total area.

[e]The bandwidth are based on re-scaled X and Y values due to uneven standard deviations.
