# Supplementary material for: Inter-nesting movements and habitat-use of adult female Kemp’s ridley turtles in the Gulf of Mexico
Source: PLoS One. 2017 Mar 20;12(3):e0174248. doi: 10.1371/journal.pone.0174248 (PMC5358874; doi:10.1371/journal.pone.0174248)
Supplement: S4 Table — (PDF) [file pone.0174248.s004.pdf]

S4 Table. Kernel density estimation (KDE), minimum convex polygon (MCP), and α-Hull results for nesting Kemp's ridley turtles (*Lepidochelys kempii*) with unsuccessful state-space model (SSM) runs.

		KDE					MCP			α-Hull		
Turtle ID #[a]	Tag no.	Band-width	50% area (km2)[c]	50% centroid depth (m)	50% centroid to land (km)	95% area (km2)[c]	Area (km2)	Centroid depth (m)	Centroid to land (km)	Area (km2)[c]	Centroid depth (m)	Centroid to land (km)
Padre Island National Seashore, Texas, USA												
P09	7681A[b]						7247.7	22	13.0	4667.4	30	23.0
P11	21811[b]						1911.4	26	16.6	1307.6	29	21.5
P14	18281[b]	2935.5	254.6	18	8.7	1518.6	6331.6	31	23.7	1343.6	24	13.5
P16	18160						2309.6	28	23.5	2309.6	28	23.5
P18	18277						901.3	20	10.9	607.6	20	10.9
P15	18308[b]	9533.3	1952.3	27	14.9	7695.1	9413.2	37	30.2	3718.6	26	14.5
P21	18277A	0.3[d]	1355.0	11	2.8	4176.8	5056.2	26	19.3	2248.5	26	18.7
P23	24857						3499.6	25	13.9	2493.6	17	7.0
P08	29351						3003.2	34	25.4	750.0	16	6.9
P20	24858[b]						4874.2	31	26.8	1415.3	20	10.2
P28	29350						5164.7	31	27.0	1499.2	19	10.2
P30	25220	0.3[d]	3518.3	22	12.6	8090.8	8748.2	31	27.7	4259.4	22	13.6
P12	25217	10189.6	2590.3	20	12.7	8609.2	7293.1	25	19.6	3238.6	16	7.4
P31	25218						4659.7	21	15.1	2477.7	20	14.6
P21	25219						4251.2	23	15.7	1331.3	16	6.7
P45	15520[b]						1551.3	25	17.3	284.0	18	9.3
P42	17804	6630.3	2100.8	17	2.7	7173.7	9088.9	26	22.6	3410.9	21	12.9
P43	17806						244.7	14	3.5	244.7	14	3.5
P44	17807						2744.5	20	9.1	2380.6	18	7.6
P55	47791	4068.5	455.7	18	7.5	2108.5	2815.0	25	20.5	540.1	17	7.0
P09	53628						1077.2	14	6.7	360.1	14	7.5
P68	53629	0.6[d]	1806.3	22	11.9	3967.1	3884.0	28	21.3	2492.0	26	16.2
P69	53630	0.2[d]	686.5	14	3.8	2606.1	4668.7	24	15.0	1339.7	19	8.4

P35	70701	0.1[d]	276.5	11	3.6	1248.1	4730.2	28	21.1	556.6	14	5.4
P104	70702[b]	3630.9	214.2	15	4.4	1287.9	1454.6	19	8.2	552.7	17	7.3
P121	70703	0.4[d]	603.4	14	5.5	1677.8	1451.4	19	9.4	832.1	17	7.3
P28	82214	0.3[d]	731.2	12	3.2	3348.7	3499.3	24	16.6	797.0	24	18.4
P92	82216	0.6[d]	867.1	15	5.1	2352.5	2694.0	28	19.7	922.3	18	7.9
P15	47529	0.2[d]	216.0	15	4.0	1528.2	3902.6	28	19.3	472.3	18	6.8
P315	47709						467.7	14	5.6	250.5	12	4.7
P280	106339						4367.5	25	19.1	932.3	17	7.3
P113	117515						3215.6	45	42.7	588.6	40	37.6
P231	117521						233.9	19	9.1	84.1	20	11.3
Rancho Nuevo, Tamaulipas, Mexico												
RN02	100397[b]						294.7	24	9.8	88.6	38	12.6
RN03	100398						865.0	20	7.5	721.0	20	7.7
RN14	100405						42.3	9	3.5	42.3	9	3.5

[a]Does not include Tag nos. 18299, 18301, and 100396 which had unsuccessful SSM runs. Although these had distance, home ranges could not be calculated so they were excluded from home range maps and this table. Of these, only 18299 passed the site fidelity test.

[b]p values for site fidelity test were > 0.95.

[c]These values include only in-water area; any land within KDE, MCP, and α-Hull contour was removed from total area.

[d]Bandwidth are based on re-scaled X and Y values due to uneven standard deviations.
