# Supplementary figures and images for: Inter-nesting movements and habitat-use of adult female Kemp’s ridley turtles in the Gulf of Mexico
Source: PLoS One. 2017 Mar 20;12(3):e0174248. doi: 10.1371/journal.pone.0174248 (PMC5358874; doi:10.1371/journal.pone.0174248)

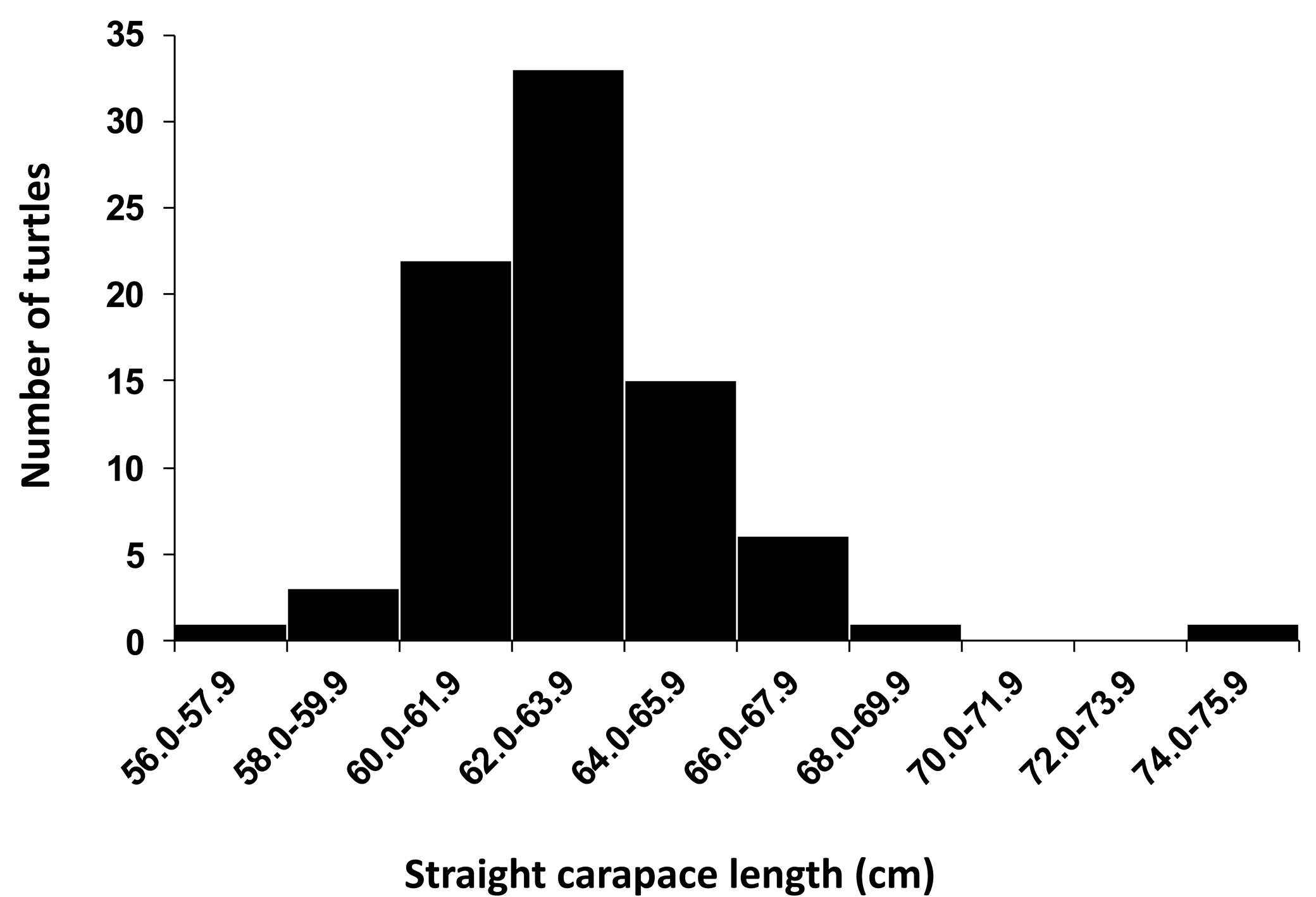

Supplement: S1 Fig — (TIF) [file pone.0174248.s005.tif]

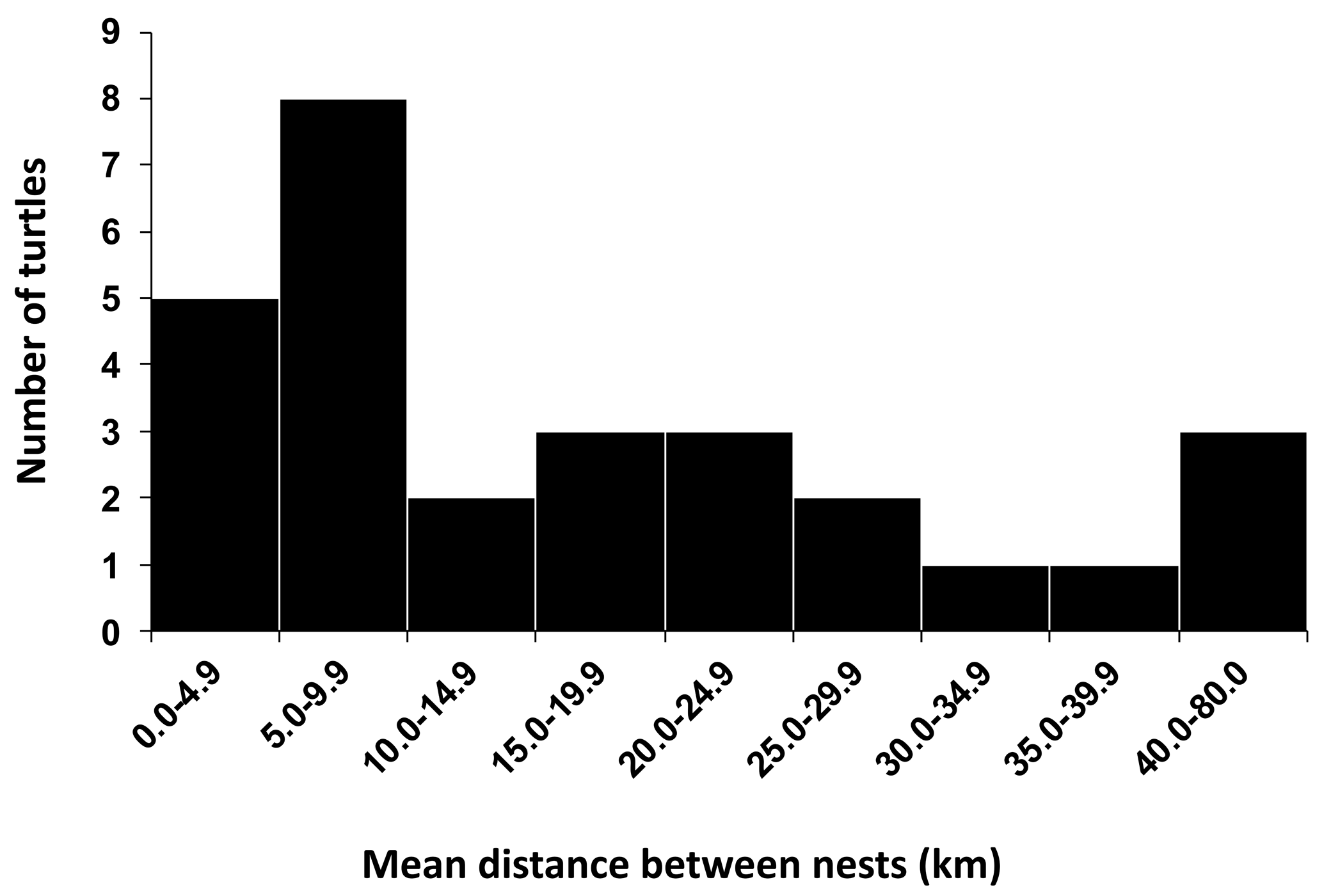

Supplement: S2 Fig — (TIF) [file pone.0174248.s006.tif]
